# Maternal-placental-fetal biodistribution of multimodal polymeric nanoparticles in a pregnant rat model in mid and late gestation

**DOI:** 10.1038/s41598-017-03128-7

**Published:** 2017-06-06

**Authors:** Diwei Ho, Joan W. Leong, Rachael C. Crew, Marck Norret, Michael J. House, Peter J. Mark, Brendan J. Waddell, K. Swaminathan Iyer, Jeffrey A. Keelan

**Affiliations:** 1 School of Molecular Sciences, The University of Western Australia, Perth, WA 6009 Australia; 2 Division of Obstetrics & Gynaecology, The University of Western Australia, Perth, WA 6009 Australia; 3 School of Human Sciences, The University of Western Australia, Perth, WA 6009 Australia; 4 School of Physics, The University of Western Australia, Perth, WA 6009 Australia

## Abstract

Multimodal polymeric nanoparticles have many exciting diagnostic and therapeutic applications, yet their uptake and passage by the placenta, and applications in the treatment of pregnancy complications have not been thoroughly investigated. In this work, the maternal-fetal-placental biodistribution of anionic and cationic multimodal poly(glycidyl methacrylate) (PGMA) nanoparticles in pregnant rats at mid (ED10) and late (ED20) gestation was examined. Fluorescently-labelled and superparamagnetic PGMA nanoparticles functionalized with/without poly(ethyleneimine) (PEI) were administered to pregnant rats at a clinically-relevant dose and biodistribution and tissue uptake assessed. Quantitative measurement of fluorescence intensity or magnetic resonance relaxometry in tissue homogenates lacked the sensitivity to quantify tissue uptake. Confocal microscopy, however, identified uptake by maternal organs and the decidua (ectoplacental cone) and trophoblast giant cells of conceptuses at ED10. At ED20, preferential accumulation of cationic vs. anionic nanoparticles was observed in the placenta, with PGMA-PEI nanoparticles localised mainly within the chorionic plate. These findings highlight the significant impact of surface charge and gestational age in the biodistribution of nanoparticles in pregnancy, and demonstrate the importance of using highly sensitive measurement techniques to evaluate nanomaterial biodistribution and maternal-fetal exposure.

## Introduction

Nanoscale drug delivery agents have been developed and exploited to enhance the delivery of pharmacologics in the treatment of a number of diseases. Despite the potential benefits of this technology in terms of pharmaceutical flexibility, selectivity, dose reduction and minimization of adverse effects, nanoparticle-mediated drug delivery agents have only been investigated in the treatment of pregnancy conditions over the past five years^1–3^. In particular, notable developments have been made in the design and use of nanomaterials to treat ectopic pregnancies and fetal growth restriction, and each of these nanomaterial platforms have unique functionalities that were advantageous to their experimental paradigm^2, 3^.

While the highly customizable nature of nanoscale-drug delivery agents is an attractive feature, the diverse array of platforms currently being investigated for biological applications also emphasizes the need to characterize each individual configuration so as to obtain a clear understanding of their biodistribution and uptake *in vivo*.

During pregnancy, the placenta is the key determinant of fetal exposure to maternally-delivered xenobiotics. A first step in developing a nano-sized maternal drug delivery agent is to study and characterize the physico-chemical properties that determine the extent to which the drug delivery agent is taken up by the placenta and passaged into the fetal circulation and organs.

In humans the placental barrier is comprised of two cellular layers: the syncytiotrophoblast membrane, which is in contact with the maternal circulation, and the endothelial layer of the fetal capillaries that is perfused by the fetal circulation^4^. The human placental structure is essentially complete by the first month of pregnancy, although villous blood perfusion and maternal-fetal exchange is not established until the end of the first trimester^5^. The rat placenta, on the other hand, does not achieve its definitive chorioallantoic structure until half way through pregnancy, and has a different zonal structure to the human placenta: a junctional zone and a labyrinth zone^6^. The latter is composed of three layers of trophoblast cells across which the majority of maternal-fetal transfer takes place^7^. Despite the structural and anatomical differences between rodent and human placentas, it is generally agreed that the functional barrier properties are similar; hence, the rodent has been widely exploited in studies of maternal-to-fetal transfer of xenobiotics and other molecules^5, 8, 9^.

Uptake and transfer of compounds and particles across the placenta can occur by passive and/or facilitated diffusion, receptor-mediated transport, and comparatively non-selective uptake processes such as pinocytosis and phagocytosis^10^. Most molecules larger than ~600 Da do not readily cross the human placenta, unless they are ligands for transporters or receptors. However, despite their relatively large size, studies have shown that some nanoparticles appear to be able to traverse the placenta and accumulate in the fetus, albeit at low levels^11–15^. The extent of transfer is also highly dependent on the size and charge of the nanoparticles^11–17^. Placental maturity is also an important factor as the fetus is highly susceptible to teratogens during early gestation^12^. In mice, Yang *et al*. reported that the uptake of gold nanoparticles in fetal tissue was markedly reduced following the establishment of the chorioallantoic placenta and haemotrophic nutrition in mid pregnancy^18^.

Due to the requirement to accurately detect fetoplacental accumulation of nanomaterials at very low levels, studies of nanoparticle biodistribution in pregnancy demand highly sensitive detection, visualization and quantitation methods. The use of multimodal labelling approaches enables synthesis of functionalized particles with improved delivery and detection capabilities and has been a widely explored subject in nanomedicine^19–21^. For example, magnetic resonance imaging (MRI) combined with fluorescence microscopy exploits the radiation-free, whole-body, deep tissue imaging ability of MRI with the sensitivity of fluorescence detection^19, 22^. Such constructs have been used to investigate nanoparticle biodistribution in experimental models of cancer and in the treatment of disease^21, 23^.

The main objective of the current study was to determine the biodistribution of both cationic and anionic multimodal (fluorescent and paramagnetic) polymeric nanoparticles in a pregnant rat model at two different stages of pregnancy (embryonic day 10 and day 20; ED10 and ED20, respectively) using pharmacological doses of nanoparticles to obtain clinically relevant information. We employed conventional whole organ imaging techniques (i.e. multispectral fluorescence imaging), quantitative measures (i.e. relaxometry and fluorometry) and confocal microscopy, finding significant limitations in sensitivity in the non-microscopic techniques. We also report the effects of surface charge and gestational age at exposure on tissue-specific localization of polymeric nanoparticles within the fetus and placenta. These findings have important implications for the development and application of multimodal polymeric nanoparticles in pregnancy.

## Results and Discussion

### Synthesis and characterization of PGMA and PGMA-PEI nanoparticles

**Figure 1:**
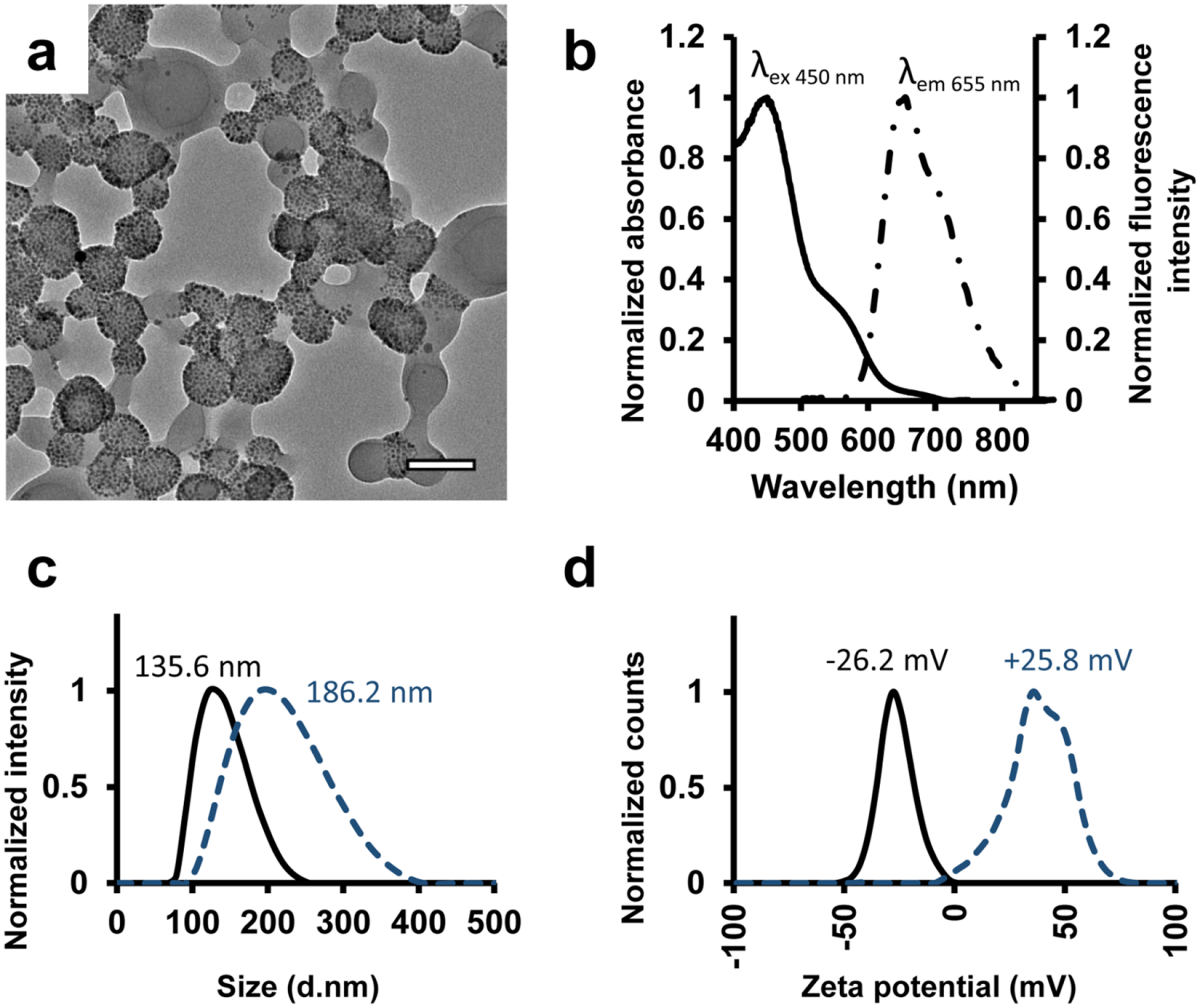
Nanoparticle characterisation. (**a**) TEM image of PGMA-PEI nanoparticles, scale bar: 100 nm. (**b**) Excitation (solid line) and emission (discontinuous line) spectra of PGMA/PGMA-PEI nanoparticles containing P10 fluorophore. (**c**) Hydrodynamic size distribution of PGMA (solid) and PGMA-PEI (discontinuous) nanoparticles. (**d**) Zeta potentials of PGMA (solid) and PGMA-PEI (discontinuous) nanoparticles.

The nanoparticles were synthesized using poly(glycidyl methacrylate) (PGMA) as the base polymer. The pendant epoxides on PGMA make it a convenient and easy-to-use platform for conjugating various functional moieties through the primary amines, thiolates and hydroxyl groups. PGMA-based nanoparticles have been used previously both *in vitro* and *in vivo*, and have shown to have good biocompatibility, low toxicity with the capability to encapsulate both therapeutic and imaging agents^24–29^. Superparamagnetic iron oxide (Fe_3_O_4_, magnetite) and a near-infrared (NIR) fluorophore known as P10 (poly[(9,9-dihexylfluorene)-co-2,1,3-benzothiadiazole-co-4,7-di(thiophen-2-yl)−2,1,3-benzothiadiazole]) were incorporated in the nanoparticle design to allow quantitation and visualization via the use of magnetic resonance relaxometry measurements and fluorescent imaging, respectively (Fig. 1a)^30^. P10 has a large Stokes shift (205 nm: λ_ex_ = 450 nm, λ_em_ = 655 nm; Fig. 1b) with emission in the NIR region, reducing interference from tissue autofluorescence and offering high tissue penetration during imaging^30^. Polyethyleneimine (PEI) was conjugated to the pendant epoxides on the polymer, producing positively-charged PGMA-PEI nanoparticles. The hydrodynamic diameters and zeta potentials (Fig. 1c and d) of the anionic PGMA (135.6 nm, PDI: 0.13; −26.2 mV) and cationic PGMA-PEI (186.2 nm, PDI 0.13; + 25.8 mV) nanoparticles were measured using the dynamic light scattering (DLS) technique. The nanoparticles were also observed to be colloidally stable in water over 7 days, with no loss in fluorescence or paramagnetic properties.

To investigate changes in the physical properties of PGMA and PGMA-PEI nanoparticles in a simulated *in vivo* setting, the nanoparticles were incubated in DMEM/F-12K cell media supplemented with fetal bovine serum (10% v/v) at 37 °C for 4 h under slow stirring. The shift in hydrodynamic diameters and zeta potentials (Fig. S1) of the PGMA (147.5 nm, PDI: 0.14; −1.4 mV) and PGMA-PEI (229.6 nm, PDI: 0.29; −4.1 mV) nanoparticles was expected and can be attributed to the formation of a protein corona and resultant charge compensation. Despite the low and negative zeta potential, corona-coated nanoparticles have been shown to be taken up by cells and delayed the cytotoxicity of cationic nanoparticles^31^. Interestingly, nanoparticles that were cationic before opsonization had increased cellular association/uptake compared to anionic nanoparticles, even after the formation of a protein corona^32, 33^.

### *In vitro* and *in vivo* assessment of nanoparticle biocompatibility

Unconjugated PEI is known to disrupt plasma membranes and induce apoptosis, resulting in cytotoxicity^34^. Furthermore, it is important to assess any interactions between nanomaterials and the immune system, as it has been shown that intrauterine inflammatory activation can alter the uptake profile of nanoparticles through the placental barrier and into fetal circulation^35^. Therefore, an *in vitro* cell viability study using human placenta choriocarcinoma cells (BeWo) was performed to assess the toxicity of the particles; no cytotoxicity was observed for either PGMA or PGMA-PEI nanoparticles over a 24 h period (Fig. S1). Next, PGMA and PGMA-PEI nanoparticles (500 µg; 1.45 ± 0.15 mg/kg) were administered intravenously to pregnant rats at ED10 and ED20 and the animals’ systemic inflammatory responses determined. None of the treated dams had detectable levels of pro-inflammatory cytokines (IL-6, IL-1β and TNF-α) in the plasma 4 h post-injection (data not presented). This suggested a lack of a systemic maternal inflammatory response to nanoparticle administration and minimal possibility of altered uptake of nanoparticles through the placental barrier.

### Quantitative assessment of nanoparticle biodistribution

To quantitatively assess the biodistribution of PGMA and PGMA-PEI within the pregnant dams, the maternal organs, conceptuses and fetuses were collected after 4 h exposure to the various nanoparticles, homogenized and assessed via magnetic resonance transverse relaxometry (R2) and fluorescence intensity (FI) measurements. The experimental endpoint at 4 h was chosen to monitor post-administration biodistribution characteristics, as the nanoparticles were expected to have a relatively short serum half-life due to the lack of ‘stealth’ moieties (e.g. PEGylation) that reduce opsonization and mononuclear phagocyte system (MPS) clearance. The 4 h endpoint should, therefore, be sufficient to assess the biodistribution of the nanoparticles over several half-lives, prior to degradation and loss of signal. This was supported by a previous study in which non-PEGylated nanoparticles of similar sizes (~230 nm) and charge (−9 mV) were found to have a half-life of 3 h^36^.

Transverse relaxation rate (R2/[Fe]) measured by relaxometry was used to quantitate tissue nanoparticle (magnetite) concentrations. Overall, most of the tissue samples lacked R2/[Fe] signals detectable above background. However, in the ED10 dams, R2/[Fe] values were significantly higher in the spleens of PGMA-PEI treated animals compared to PGMA treated (n = 4; p = 0.029) and control animals (n = 4; p = 0.023), although this finding was not mirrored in the corresponding tissue samples at ED20 (Fig. S2). We concluded that relaxometry lacked the necessary sensitivity for detecting significant amounts of nanoparticles in maternal, placental and fetal tissues (Fig. S2).

**Figure 2:**
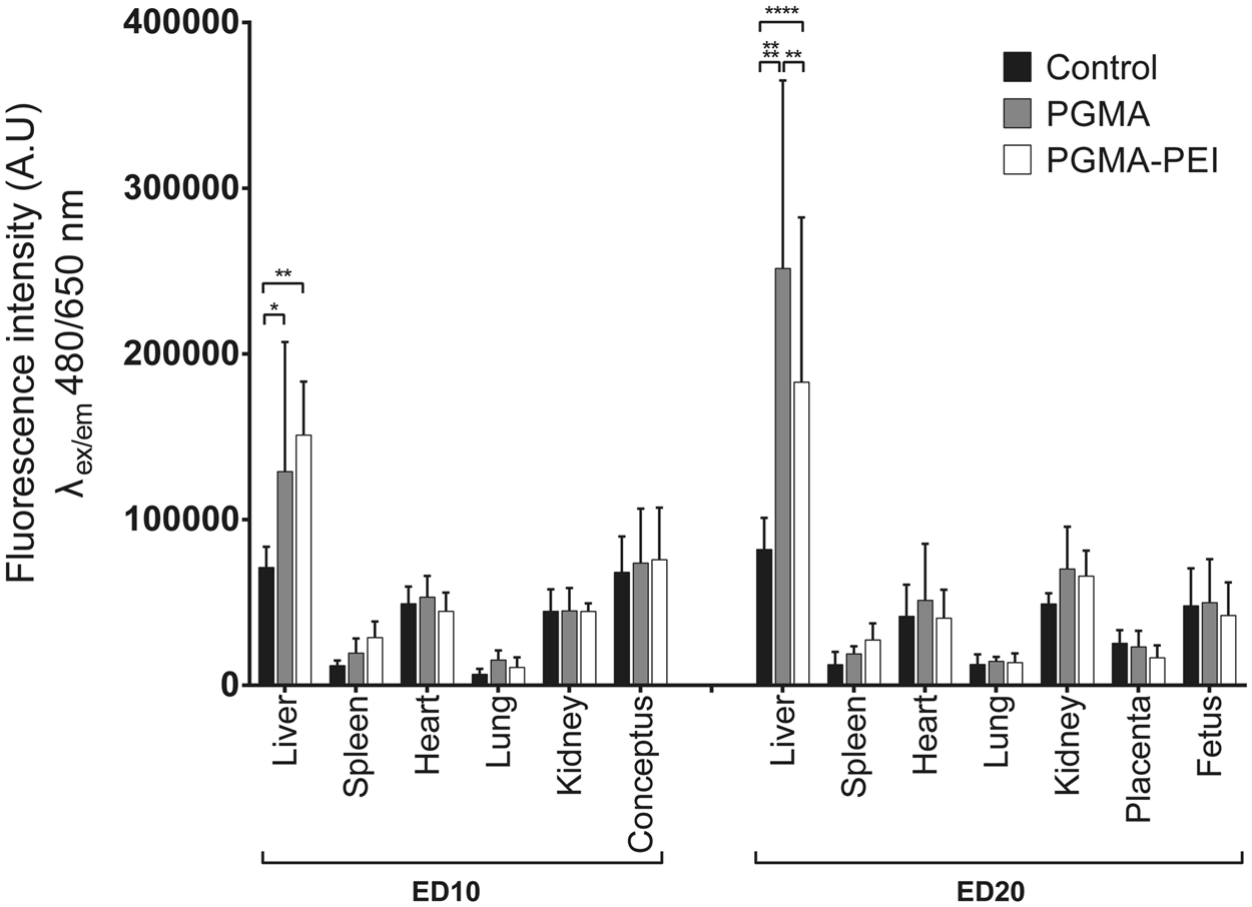
Fluorescence intensity measurements of ED10 and ED20 tissue homogenates. Data reported as mean ± SD (n = 4 per group). One-way ANOVA was used for statistical analysis; *p ≤ 0.05; **p ≤ 0.01; ****p ≤ 0.0001.

As an alternative detection strategy, P10 fluorescence was measured in tissue homogenates; data were normalized to tissue weight. Significantly higher P10 fluorescence levels were observed in liver homogenates from ED10 and ED20 dams following PGMA and PGMA-PEI administration relative to controls (Fig. 2) reflecting nanoparticle accumulation in hepatic tissue. In ED10 dams, PGMA and PGMA-PEI fluorescence intensities were comparable within the liver samples. However, at ED20, fluorescence signals from animals exposed to PGMA nanoparticles were significantly higher (~1.4 fold, p = 0.0026) compared to PGMA-PEI particles. Contrary to results obtained from relaxometry measurements (Fig. S2), there was no significant difference in the amount of P10 fluorescence arising from the spleen homogenates from PGMA and PGMA-PEI treated ED10 dams. Hemoglobin in blood can quench fluorescence signals in the NIR region; thus it is possible that the P10 signal may have been quenched by the high hemoglobin content within the spleen homogenates^37^. Tissue quenching of Cy5.5 and Texas Red fluorescence has also been previously described in the liver and spleen^37^. In addition, there were no significant differences between the same treatment groups at the two gestational ages. Despite the large Stokes shift of P10 and its NIR emission spectrum, broad spectrum autofluorescence from homogenates remained problematic, particularly in the placental samples. This autofluorescence, combined with absorbance from hemoglobin derivatives, could have obscured P10 fluorescence signal and contributed to the relative lack of sensitivity^38^. This was consistent with the high levels of intrinsic autofluorescence observed in the heart^39^.

### Qualitative assessment of nanoparticle distribution using ex vivo fluorescence imaging

**Figure 3:**
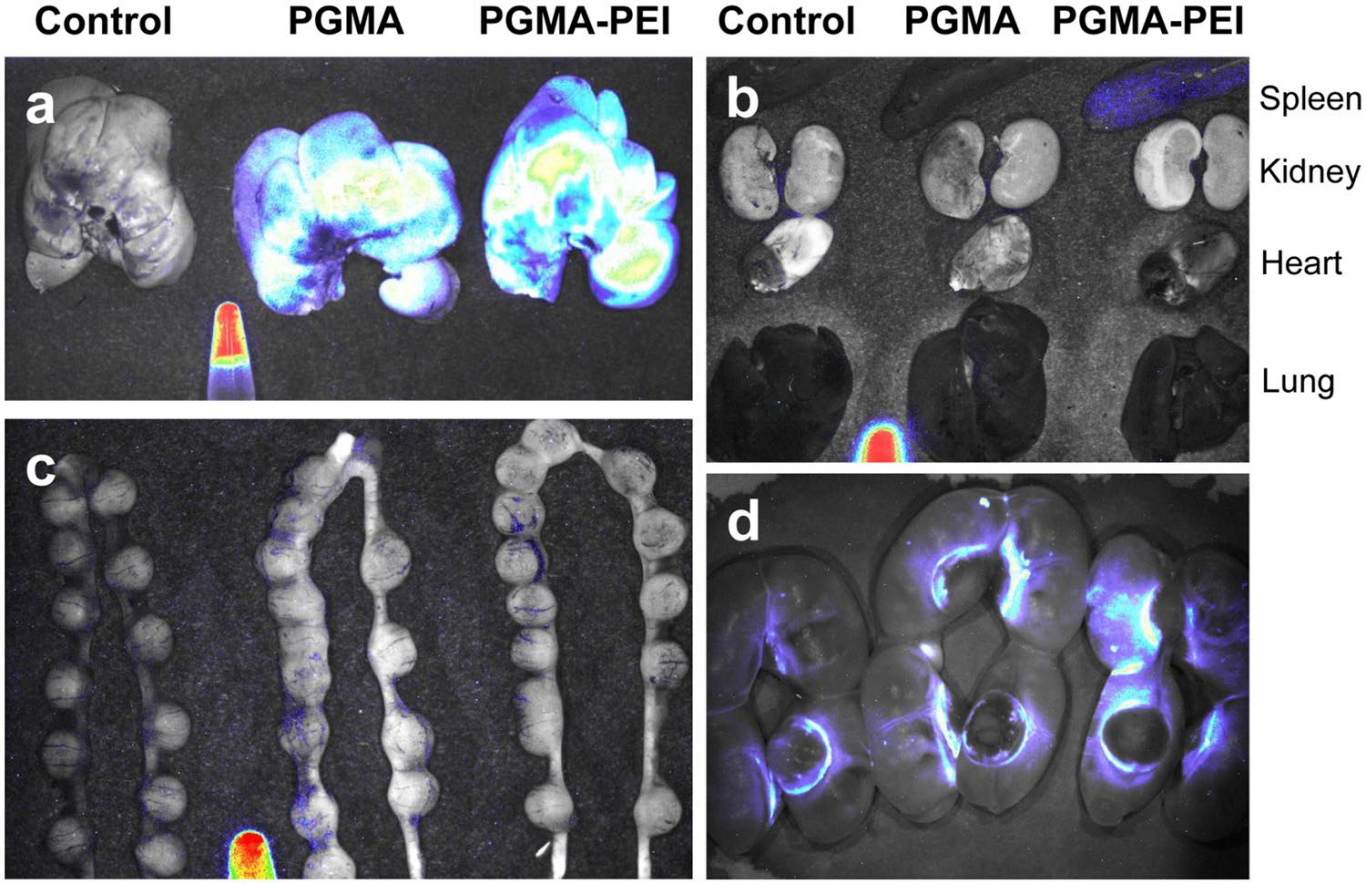
Whole tissue fluorescence imaging of representative ED10 and ED20 tissues. Spectral unmixing was performed using a tube containing PGMA-PEI nanoparticles as reference. (**a**) Livers of ED10 dams. High levels of fluorescence were observed in livers from both PGMA and PGMA-PEI treated dams. (**b**) Other organs of ED10 dams. With the exception of the spleen of the PGMA-PEI treated dam, none of the organs showed a visible level of P10 fluorescence. (**c**) Uterine horns containing conceptuses of ED10 dams. In spite of spectral unmixing, low levels of autofluorescence was observed in controls and treated dams. (**d**) Uterine horns containing placentae and fetuses of ED20 dams. The placentae showed a visible level of autofluorescence in the P10 spectral region around the junctional zones in all dams. Images are representative of n = 4 per group.

Organs excised 4 h post-treatment were imaged *ex vivo* using a whole animal multispectral imaging system to assess the gross anatomical distribution of P10-labelled nanoparticles within the organs. Spectral unmixing (using PGMA-PEI nanoparticles as reference spectra) was performed in order to selectively remove autofluorescence. However, due to the low levels of nanoparticles in the tissues, P10 fluorescence was not readily observed in most organs, except in the livers of PGMA/PGMA-PEI treated dams and spleens of PGMA-PEI treated dams (Fig. 3a and b). These observations were congruent with the trends seen in the quantitative fluorescence measurements.

In spite of the use and advantages of spectral unmixing, significant autofluorescence was observed in the uterine horns containing conceptuses of ED10 controls and treated dams (Fig. 3c). In each group of the ED20 dams, the placentae showed bright autofluorescence in the P10 spectral region around the periphery of the decidual cap, possibly representing the parietal yolk sac (Fig. 3d). It is possible that the autofluorescence observed in the placentae was a result of accumulation of refractile bodies and lipofuscin-like fluorescent pigments (λ_ex = _320–480 nm, λ_em_ = 460–630 nm) which overlap with the spectral profile of P10 and so decreased the effectiveness of spectral unmixing^40, 41^. This is further detailed in the following section.

### Qualitative assessment of nanoparticle distribution using confocal imaging

Tissue samples were fixed and cryosectioned for confocal imaging to observe nanoparticle distribution within the tissue structures. Gross structural morphology of the tissue was observable by autofluorescence (λ_ex_ = 488 nm, λ_em_ = 500–525 nm) without further staining. P10 fluorescent signals were visualized at 663–738 nm. In contrast to the lack of definitive measurements of nanoparticle uptake by R2/[Fe] and fluorescence measurements, differences in nanoparticle abundance between samples from nanoparticle-treated versus control dams were clearly observed using confocal microscopy. Certain cellular components, such as refractile bodies and lipofuscin-like fluorescent pigments, can be misclassified as fluorescent nanoparticles^42^. However, although very similar in morphology to nanoparticle aggregates, refractile bodies were easily differentiated from the PGMA and PGMA-PEI nanoparticles under confocal imaging. Refractile bodies appeared in both autofluorescence (500–525 nm) and P10 (663–738 nm) collection channels, while nanoparticles appeared only in the P10 channel during imaging. This particular property of P10 has distinct advantages over the yellow-green fluorescent nanoparticles (λ_em peak_ = 511 nm) used by others, in that specific signals can be visualized in the tissues^14^. Strong, punctate autofluorescent signals arising from refractile bodies were observed in tissues such as the hearts and conceptuses. Nanoparticles were normally observed as aggregates; the smallest observable nanoparticles in the confocal images were approximately 650 nm in size.

Fluorescent nanoparticle accumulation was readily observable microscopically within the maternal liver (Fig. S3), spleen (Fig. S4) and lungs (Fig. S5), and to a lesser extent in the heart and kidney (data not shown). Nanoparticles were more readily apparent in the PGMA-PEI-treated dams compared to PGMA-treated dams. Within the liver, PGMA-PEI and PGMA nanoparticles were found mainly within Kupffer cells in the hepatic sinusoids of the liver, in close proximity to the central veins of the lobules. In the spleen, nanoparticles were evenly distributed within the red pulp, with higher quantities amassing around the peripheries of white pulp (marginal zone). In contrast, few or no nanoparticles were seen within the white pulp, trabeculae and capsule. Nanoparticles were readily observed in the maternal lungs, evenly distributed in the alveolar lining with little to none observed within the bronchioles and blood vessel endothelium. The detection of PGMA and PGMA-PEI nanoparticles within the liver, spleen and lungs was in line with other biodistribution studies using different systems^43^. The liver, spleen and lungs, being part of the MPS, typically sequester large nanoparticles in high quantities as a consequence of recognition and phagocytosis by macrophages^44^.

Nanoparticles were only occasionally seen interspersed within the myocardium and the endocardium of the maternal heart, and no particles were observed in the epicardium. Likewise, nanoparticles were only sparsely distributed in the kidneys. Within the renal cortex, nanoparticles were more readily found in renal corpuscles than the surrounding tissue. Overall, in maternal organs there were no apparent differences in uptake between ED10 and ED20 dams. This has also previously demonstrated with surface-functionalized gold nanoparticles in mice^45^.

**Figure 4:**
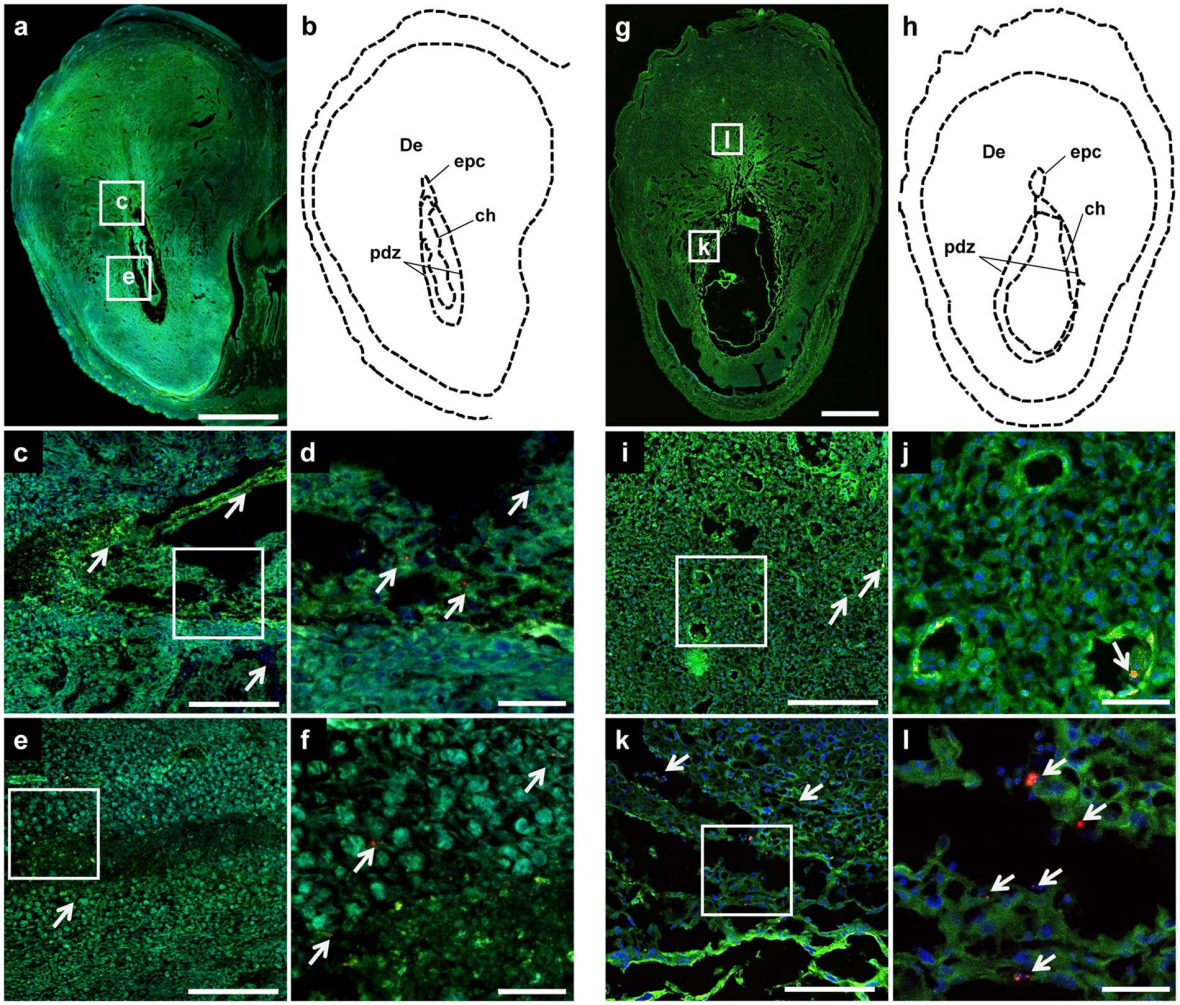
(**a**–**f**) PGMA and (**g**–**l**) PGMA-PEI nanoparticles in the ED10 conceptus. (**a**) Representative tissue section of a conceptus from a PGMA treated dam. (**b**) Outline of section denoting tissue regions. (**c**–**e**) Higher magnification images of the areas shown in the rectangles in (**a**). (**d**,**f**) High magnification images of panels (**c**) and (**e**) respectively. PGMA nanoparticles (indicated by arrowheads) were observed in the ectoplacental cone as well as in the primary decidual zone. (**g**) Representative tissue section of a conceptus from a PGMA-PEI treated dam. (**h**) Outline of section denoting tissue regions. (**I**,**k**) Higher magnification images of the areas shown in the rectangles in (**g**). (**j**,**l**) High magnification images of panels (**i**) and (**k**) respectively. PGMA-PEI nanoparticles (indicated by arrowheads) were observed in the decidua, specifically in the venous sinusoids and tissue close to the ectoplacental cone as well as in the primary decidual zone. Scale bars: (**a**,**g**) 1000 µm; (**c**,**e**,**i**,**k**) 200 µm; (**d**,**f**,**j**,**l**) 50 µm. De: decidua; ch: chorion; epc: ectoplacental cone; pdz: primary decidual zone. Images are representative of n = 4 per group.

At ED10, developing conceptuses were sectioned without separation of the placenta and fetus as these were not readily distinguishable. Nanoparticles were observed in the conceptuses from both PGMA and PGMA-PEI treated dams (Fig. 4). While the observed nanoparticles were relatively sparse compared to the other major organs examined in this study, PGMA and PGMA-PEI nanoparticles were found to be dispersed throughout the decidua and present within the ectoplacental cone (Fig. 4C, D, I and J) and the primary decidual zone, more specifically within the trophoblast giant cells (TGCs; Fig. 4e, f, k and l). The ectoplacental cone is thought to differentiate into spongiotrophoblasts which eventually differentiate into glycogen trophoblast cells (GTCs) - one of the four subtypes of trophoblast giant cells^46, 47^. The role of GTCs is still not fully elucidated, but they are speculated to assist in implantation and/or to act as an energy source for the placenta or the fetus via the metabolism of glycogen stores into glucose^48, 49^. Additionally, GTCs secrete a host of proteins (e.g. extracellular matrix, cytokines and hormones) that are proposed to be involved in the implantation and the maintenance of pregnancy^47^. To the best of our knowledge, this is the first time that nanoparticles have been localized to specific tissues in a developing rat conceptus this early in gestation. It is difficult to predict the consequences of this exposure within a developing conceptus, but this represents a challenge and direction for future studies.

**Figure 5:**
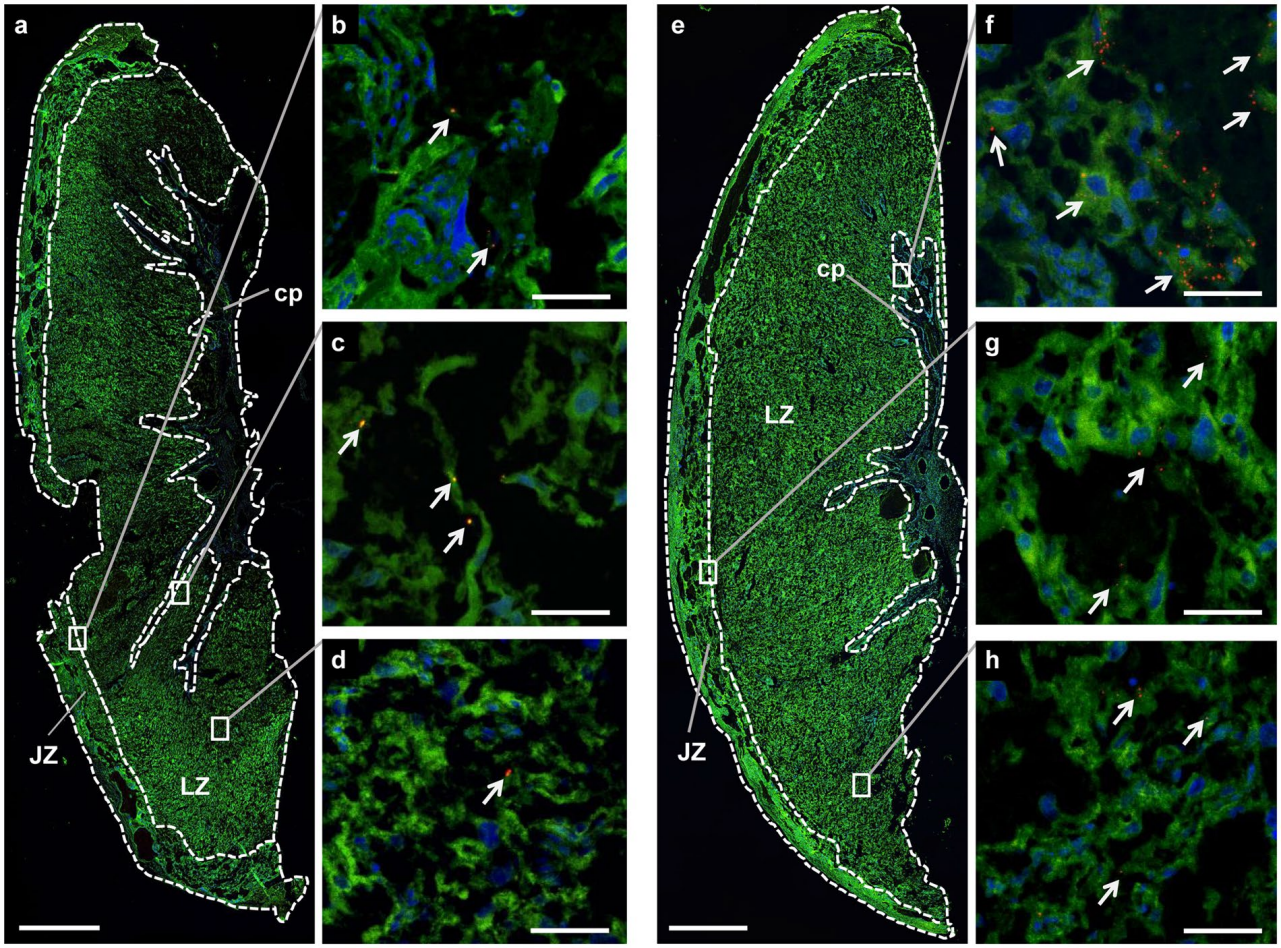
(**a**–**d**) PGMA and (**e**–**h**) PGMA-PEI nanoparticles in the placenta at ED20. (**a**) Representative tissue section of a placenta from a PGMA treated dam with key tissue regions indicated (n = 4 per group). (**b**–**d**) Higher magnification images of the areas indicated by the rectangles in (**a**). PGMA nanoparticles (indicated by arrowheads) were observed in the (**b**) chorionic plate, in the tissue adjacent to the (**c**) junctional zone and in the (**d**) labyrinth zone. (**e**) Representative tissue section of a placenta from a PGMA-PEI treated dam with key tissue regions indicated (n = 4 per group). (**f**–**h**) Higher magnification images of the areas indicated by the rectangles in (**e**). PGMA-PEI nanoparticles (indicated by arrowheads) were observed in the (**f**) chorionic plate, in the tissue adjacent to the (**g**) junctional zone and in the (**h**) labyrinth zone. Scale bars: (**a**,**e**) 1000 µm; (**b–d,f–h**) 200 µm. JZ: junctional zone; LZ: labyrinth zone; cp: chorionic plate.

At ED20 conceptuses were separated into the placenta and fetus. PGMA nanoparticles were observed within treated placentae (Fig. 5a–d), but were very sparse and tended to appear as singular aggregates rather than the clusters observed in placentae from PGMA-PEI treated dams (Fig. 5e–h). Lipofuscin-like fluorescent pigments and refractile bodies were observed in high concentration around the decidual caps (Fig. S6). Though PGMA nanoparticles were evenly distributed across the two zones of the placentae, this was at a significantly lower average density of nanoparticles per mm^2^ of tissue section (14.2 ± 2.4, n = 12; Fig. S7). Nanoparticles were seen at a greater frequency in the placentae of dams treated with PGMA-PEI nanoparticles, with the average number of nanoparticle clusters observed being 114.9 ± 29.2 per mm^2^ of tissue section (n = 12; Fig. S7). PGMA-PEI nanoparticles were observed most commonly within the labyrinth zone at the chorionic plate (Fig. 5f; Fig. S8). These findings obtained from confocal imaging suggest that PGMA-PEI nanoparticles showed preferential uptake in the rat placenta, a result not detectable using relaxometry and fluorescence intensity measurements.

Conflicting results have previously been presented with respect to the polarity of surface charge and uptake in the placenta, but it is important to note that these studies were performed in different experimental models. Specifically, Di Bona and colleagues reported that cationic nanoparticles preferentially accumulate within the murine placenta at high doses, but Bajoria *et al*. showed that anionic liposomes had increased uptake by the human placenta^15, 17^. Interestingly, many studies examining uptake and/or transfer have utilized only negatively charged nanoparticles, while positively charged nanoparticles have not received as much attention^18, 45, 50–52^. Additional work will be required to establish the relationship between the charge polarity of nanoparticles, their associated protein coronas and species-specific uptake (i.e. human, rat, mouse) in the placenta.

The fetal livers, spleen and lungs were carefully analyzed to assess the presence of nanoparticles; no nanoparticles were observed within the ED20 fetuses, irrespective of treatment (data not shown). If there was any nanoparticle accumulation in the fetal organs, it was below the level of detection and visualization. While nanoparticles were observed around the syncytium of the placenta in PGMA-PEI treated animals, there was no sign of nanoparticle accumulation within the fetuses, suggesting that the extent of transplacental passage of PGMA-PEI nanoparticles is very low at this stage of gestation. In contrast, others have observed significant accumulation of larger silicon nanoparticles (519 nm) within the fetal and uterine tissue, but not within the placenta, further emphasizing that the core material of the nanoparticles, plus size and charge polarity, all play pivotal roles in biodistribution during pregnancy.

## Conclusions

Biocompatible polymeric materials such as PGMA have been investigated as delivery platforms for therapeutics due to their adaptability and utility, and have not shown toxicity *in vivo* or *in vitro* ^24, 26^. The versatility of these materials allows inclusion of labelling components like fluorescent dyes and/or magnetite for the development of a system with multimodal imaging and drug delivery capabilities^25^. Although such polymeric nanoparticles have been widely investigated across various medical applications, they have not been exploited in the context of drug delivery in pregnancy. As a prelude to further therapeutic studies, we demonstrated that analysis of biodistribution of polymeric nanoparticles at pharmacological doses in a pregnant rat model, via techniques such as relaxometry and multispectral imaging, provided a relatively insensitive representation of tissue uptake in the pregnant rat. In contrast, while the aforementioned techniques were unable to clearly determine tissue uptake quantitatively, analysis of uptake via confocal microscopy confirmed a differential charge-based accumulation of these nanoparticles in the rat placenta, with cationic nanoparticles exhibiting greater accumulation within the chorionic plate than anionic particles. It has been reported that, in general, smaller particles exhibit greater placental uptake and passage than larger particles; the increased placental uptake of our PEI-PGMA nanoparticles is, therefore, likely to be in spite of their larger size, not because of it. Additionally, nanoparticles were also observed to accumulate in different compartments of the conceptus during early, but not late, gestation, consistent with the lack of a developed placental barrier during early gestation. Further work will need to be performed using more sensitive detection and quantitation modalities to elucidate the extent/effects of nanoparticle accumulation within these particular regions and the impact of exposure time, clearance and dose.

This work highlights the advantages and limitations of the use of multimodal nanoparticles to accurately measure the uptake of polymeric nanoparticles and subsequently the extent and impact of maternal, fetal and placental exposure. Furthermore, the findings reported in this study expand our understanding of the biodistribution of charged polymeric nanoparticles and their association within the different maternal, placental and fetal compartments. This knowledge will facilitate the development of future nanomaterial-based targeted therapeutics for the treatment and management of complicated pregnancies.

## Methods

### Materials

All reagents were purchased from Sigma-Aldrich and used as received unless otherwise stated: glycidyl methacrylate (GMA, 97%), 2,2′-azobis(2-methylpropionitrile) (AIBN, 0.2 M in toluene), benzyl ether (99%), iron(III) acetylacetonate (97%), oleic acid (BDH, 92%), oleylamine (70%), 1,2-tetradecanediol (90%), Pluronic^®^ F-108 (M_n_ = 14,600 g mol^−1^), methyl ethyl ketone (MEK; Fischer Chemical, ≥99%), poly(ethyleneimine) (PEI, branched, 25 kDa).

### PGMA and PGMA-PEI nanoparticle synthesis

Poly(glycidyl methacrylate) (PGMA, M_w_ = 454,270, PDI = 1.79) was synthesized using a free-radical polymerization process. GMA was dissolved in MEK and using AIBN as an initiator, the reaction was heated to reflux under an inert atmosphere. The mixture was allowed to cool and PGMA was precipitated in methanol and collected by filtration.

P10 (PFBTDBT10) and magnetite nanoparticles were synthesized as previously described by Ding *et al*. and Sun *et al*. respectively^30, 53^. In brief, PGMA nanoparticles were synthesized using the oil-in-water emulsion method. The organic phase was prepared by dispersing magnetite nanoparticles (30 mg) and dissolving PGMA (300 mg) and P10 (10 mg) in a 1:1:2 mixture of CHCl_3_:THF:MEK. This organic phase was added dropwise to a rapidly stirring aqueous solution of the surfactant Pluronic^®^ F-108 (1.25% w/v) and the emulsion was homogenised with 20 kHz probe-type ultrasonicator at 4W_rms_ for 1 min. Organic solvents were removed under reduced pressure at 40 °C resulting in a translucent orange suspension. The suspension was collected and centrifuged at 3000 × g for 45 min to remove large aggregates, leaving colloidally stable nanoparticles in the supernatant. This supernatant was subsequently loaded onto a magnetic separation column (LS; Miltenyi Biotec) in the presence of a rare earth magnet. Nanoparticles containing magnetite were retained within the column while nanoparticles without magnetite were washed through the column with Pluronic^®^ solution. Magnetic PGMA nanoparticles were eluted in the absence of the magnet with Pluronic^®^ solution.

A portion of the collected PGMA nanoparticles was used for functionalization with PEI. The suspension was added to an aqueous solution of PEI (1 mg/ml) under vigorous stirring and heated to 70 °C for 18 h. The suspension was then centrifuged at 24,000 × g for 20 mins and the supernatant containing unreacted PEI and other unencapsulated material was removed. The pellet containing the PGMA-PEI nanoparticles was resuspended in ultrapure water and the wash step repeated. These wash steps were also performed on PGMA nanoparticles prior to use. Concentrations of both the PGMA and PGMA-PEI suspensions were determined by measuring the mass of the lyophilized sample from a known volume in a pre-weighed tube.

### Nanoparticle characterization

The hydrodynamic diameters and zeta potential of PGMA and PGMA-PEI nanoparticles were first assessed using dynamic light scattering technique (DLS; Nano ZS, Malvern Instruments, UK) at a temperature of 25 °C in deionised water and measured in triplicate. To simulate an *in vivo* setting, 500 µg of PGMA or PGMA-PEI nanoparticles were incubated in 10 ml of DMEM/F-12K medium (ThermoFisher Scientific, Australia) supplemented with fetal bovine serum (10% v/v; ThermoFisher Scientific) at 37 °C for 4 h with gentle stirring. After incubation, aliquots of the suspension were left to cool to room temperature and measured using DLS at 25 °C in DMEM/F-12K medium. Spectral response of the nanoparticles were determined using the Cary Eclipse fluorescence spectrophotometer (Agilent Technologies). For transmission electron microscopy, PGMA-PEI nanoparticles were drop-casted on carbon-coated copper TEM grids and imaged at an accelerating voltage of 120 kV on a JEOL 2100 transmission electron microscope.

### Animals

Nulliparous albino Wistar rats, 8 to 12 weeks old, were obtained from the Animal Resources Centre (Murdoch, Australia) and housed under a standard 12 h light/dark cycle and fed standard rat chow and water *ad libitum*. Procedures conformed to “Principles of Laboratory Animal Care” (NIH publication No. 86–23, revised 1985) and were approved by The University of Western Australia’s Animal Ethics Committee (RA/3/100/1200). Rats were mated overnight, with day 1 of pregnancy designated as the day on which spermatozoa were present in a vaginal smear. Studies used n = 4 dams per group unless otherwise stated.

### Nanoparticle administration and tissue collection

Prior to administration to the dams, PGMA and PGMA-PEI nanoparticle formulations were diluted to 0.5 mg/ml in saline and briefly sonicated to ensure an even suspension. At day 10 (ED10) or day 20 (ED20) of gestation, rats were anesthetized with isoflurane/nitrous oxide (70:30) and 1 ml (1.45 ± 0.15 mg/kg dose) of either PGMA or PGMA-PEI nanoparticles (0.5 mg/ml) were administered via tail vein injection. Control groups were injected with 1 ml of saline. The average weights for ED10 and ED20 dams were 313 g and 394 g (1.45 ± 0.15 mg/kg dose), respectively. The rats were returned to their housing units and were anesthetized and euthanized by pentobarbital sodium 4 h later. Maternal organs including the liver, spleen, kidney, heart, lungs, conceptuses (ED10 only), placentae (ED20 only) and fetuses (ED20 only) were excised with ceramic scalpel blades to avoid iron contamination and apportioned for analyses (i.e. relaxometry, fluorescence intensity measurements, inductively coupled plasma atomic emission spectroscopy (ICP-AES) and confocal microscopy). Conceptuses (ED10) or fetuses (ED20) were collected from the distal ends (one from each end) and two from the central region of the uterine horns to a total of four conceptuses/fetuses per dam. Maternal blood samples were obtained through cardiac puncture and centrifuged at 13,000 g for 10 min to obtain plasma. Plasma samples were frozen in stored at −80 °C until further analysis. A portion of the tissue samples were rinsed with phosphate buffered saline (pH 7.2) to remove excess blood, weighed and then homogenized with ceramic beads (PRECELLYS® 24, Bertin Technologies) as per recommended manufacturer’s instructions for fluorescence intensity measurements, relaxometry studies and ICP-AES.

### ELISA inflammatory cytokine assays

Levels of the pro-inflammatory cytokines TNF-α, IL-1β and IL-6 and were measured in maternal plasma using commercially obtained ELISA kits (TNF-α Rat ELISA Kit #ab46070, Abcam; IL-1β Rat ELISA Kit #ab100767, Abcam; IL-6 Quantikine Rat ELISA Kit #R6000B, R&D Systems) and performed as per manufacturer’s instructions. Limits of detection for the ELISA kits were: TNF-α = 31.3 pg/ml; IL-1β = 68.6 pg/ml; IL-6 = 62.5 pg/ml.

### Fluorescence intensity measurements

Homogenized tissue samples were aliquoted (100 µl) into 96-well plates (Greiner CELLSTAR®, Sigma-Aldrich) in triplicate and measured using a fluorescence plate reader (Enspire Multimode plate reader, PerkinElmer) at an excitation λ of 480 nm and collection λ of 650 nm.

### *Ex vivo* whole tissue imaging

Collected maternal organs and fetal tissues from ED10 and ED20 dams were imaged using the Maestro 2 multispectral imaging system (Cambridge Research & Instrumentation Inc.; Centre for Microscopy, Characterisation and Analysis, The University of Western Australia). Acquisition settings were set at an excitation λ of 435–480 nm, collection λ of 500–720 nm and exposure of 1000 ms. Spectral unmixing was performed using a tube of PGMA-PEI nanoparticles as reference spectra to reduce autofluorescence in the images.

### Confocal microscopy

The tissue samples were excised and post fixed in 4% paraformaldehyde overnight at 4 °C and then replaced in a sucrose gradient solution, from 15% to 30% over 48 h to ensure complete impregnation of tissue. Prior to sectioning, the tissue samples were blocked and frozen in cryo-embedding compound (Pelco®, Ted Pella). Maternal liver, heart, lung, kidney, spleen and placental tissue sections were cut at a thickness of approximately 7 µm. The ED10 conceptus and ED20 fetal tissue sections were cut at 10 µm and 12 µm, respectively. All tissue sections were cut on a cryostat (Leica CM 3050) and stored at −80 °C prior to further analysis. Cryosections were imaged by confocal microscopy (Nikon A1Si, Centre for Microscopy, Characterisation and Analysis, The University of Western Australia). The gross morphology of the tissue was imaged by autofluorescence (excitation λ = 488 nm; emission λ = 500–525 nm). Due to the large Stokes shift for the P10 fluorophore, the fluorescence signal (excitation λ = 488 nm; emission λ = 663–738 nm) was captured with a significant reduction of interference from tissue autofluorescence. DAPI (4′,6-diamidino-2-phenylindole; DNA stain) was imaged with an excitation λ of 405 nm and collection λ of 425–475 nm. Images were captured at 20x magnification and analysis was performed on 2–4 whole sections per tissue per treatment group for each gestational date. No fluorescence from nanoparticles were observed in tissue samples (maternal organs, conceptus, placentae and fetus) from control samples (data not shown). The minimum sizes of the nanoparticle aggregates were determined from the shortest resolvable distance between two confirmed nanoparticle signals. Nanoparticle counts for the PGMA and PGMA-PEI placentae sections (4 dams per group, 3 sections each) were analyzed using the open-source image processing package, FIJI^54^. From the raw images, the images of the autofluorescence channels and P10 channels were extracted. Next, the P10 channel image had its corresponding autofluorescence channel image subtracted to yield a resultant image with nanoparticle-only signals. Thresholding was applied using appropriate levels to the image to further reduce noise before doing an automated particle count using FIJI.

### TEM tissue preparation and imaging

Portions of paraformaldehyde-fixed ED20 maternal liver and placenta (labyrinth zone) were obtained and processed for TEM by post fixing in 1% osmium (ProSciTech, Australia). This was followed by dehydration of the tissue through an ethanol series to propylene oxide, infiltration and embedding into Procure-Araldite epoxy resin (ProSciTech). The epoxy resins were cured for 24 h and subsequently sectioned at 150 nm thickness onto copper TEM grids. TEM imaging was performed at an accelerating voltage of 120 kV on a JEOL 2100 transmission electron microscope.

### Statistical analyses

Data are presented as mean ± SD. Results from magnetic resonance relaxometry and fluorescence intensity measurements were subject to statistical analyses by 1-way ANOVA with Tukey’s multiple comparisons test, while differences in mean nanoparticles counts in the placental confocal images was statistically tested by 2-tailed Student’s t-test. These analyses were performed using the Prism 6 statistical package (GraphPad Software). Comparisons were considered to be statistically significant at *p* < 0.05.

## Electronic supplementary material

Supporting Information
