# Supplementary material for: Maternal-placental-fetal biodistribution of multimodal polymeric nanoparticles in a pregnant rat model in mid and late gestation
Source: Sci Rep. 2017 Jun 6;7:2866. doi: 10.1038/s41598-017-03128-7 (PMC5460222; doi:10.1038/s41598-017-03128-7)
Supplement: Supplementary file 1 — Supporting Information [file 41598_2017_3128_MOESM1_ESM.doc]

Supporting Information

**Maternal-placental-fetal biodistribution of multimodal polymeric nanoparticles in a pregnant rat model in mid and late gestation**

Diwei Ho‡, Joan W. Leong‡, Rachael C. Crew, Marck Norret, Michael J. House, Peter J. Mark, Brendan J. Waddell, K. Swaminathan Iyer* & Jeffrey A. Keelan*

Supplemental experimental section

*Cell culture:* Human placenta choriocarcinoma cells (BeWo) was obtained from ATCC (CCL-98) and were cultured in T75 cell culture treated flasks with filter caps (ThermoFisher Scientific) in a humidified atmosphere containing 5% CO_2_ at 37°C, and maintained in DMEM/F-12K medium further supplemented with fetal bovine serum (10% v/v) and GlutaMAX™ (ThermoFisher Scientific)

*Cell viability assays:* Cell viability was measured using a LIVE/DEAD® cytotoxicity kit (ThermoFisher Scientific). BeWo cells were incubated for 24 h in 6-well plates before cell media was replaced with PGMA or PGMA-PEI nanoparticle suspensions (0, 10 or 100 µg/ml final concentration of nanoparticles, n=3 per treatment group) made up in fresh cell media. The cells were further incubated at 5% CO_2_, 37°C for 4 or 24 h before removal of nanoparticle suspensions and washing with PBS (pH 7.2). The cells were treated with LIVE/DEAD® reagents according to manufacturer’s protocol and imaged on an inverted fluorescence microscope (Olympus IX71). Three images were recorded from each well at consistent locations for all wells at 10x magnification. Cell viability was represented by the proportion of live cells over the total cell count (both live and dead) and normalized to the control condition (0 µg/ml nanoparticle concentration) for each time point.


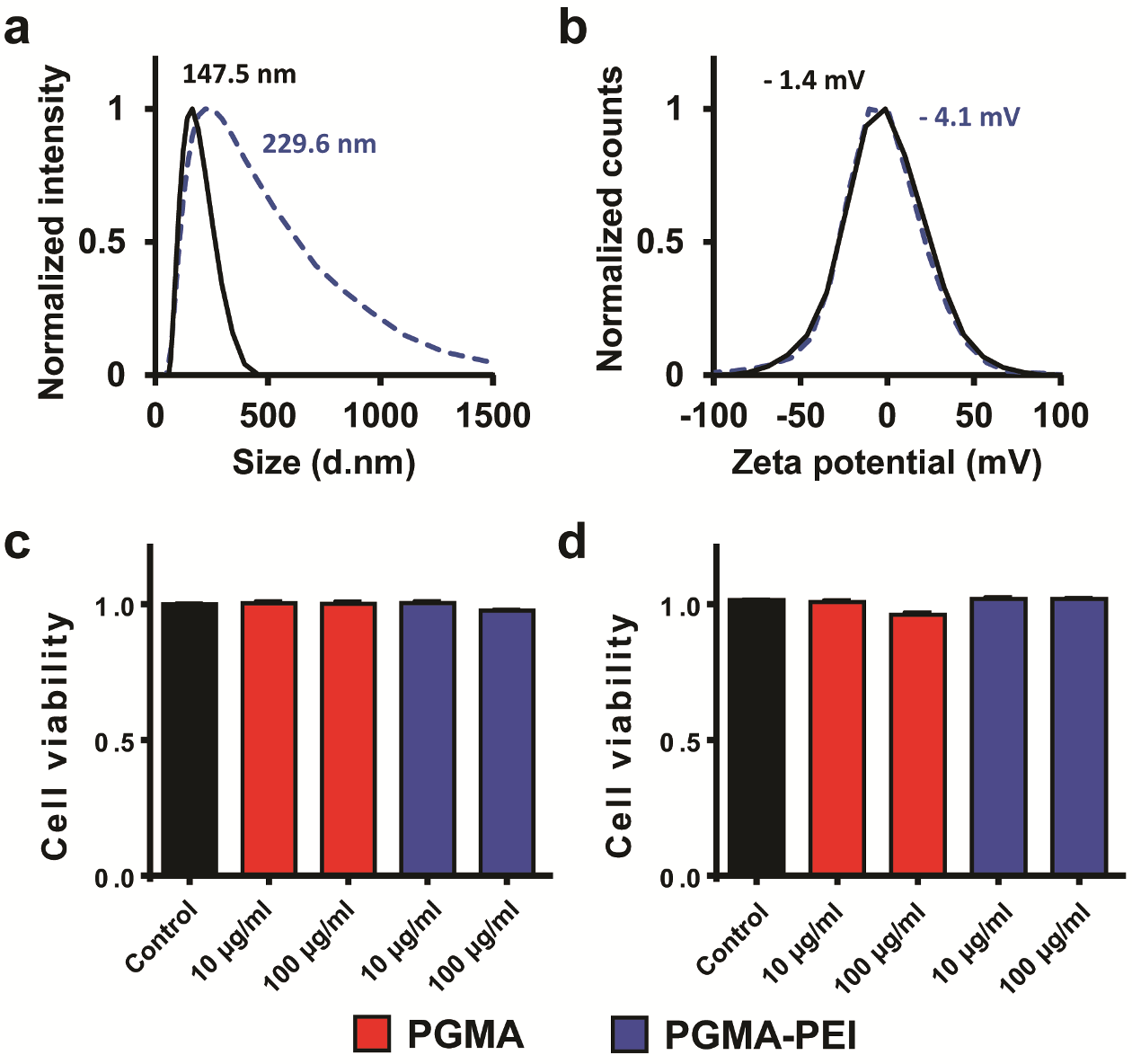


**Figure S1.** (a) Hydrodynamic size distribution and (b) zeta potentials of PGMA (solid) and PGMA-PEI (discontinuous) nanoparticles after incubation in serum-supplemented media for 4 h. Cytotoxicity assays of PGMA (red bars) and PGMA-PEI (blue bars) at 10 and 100 μg/ml after (c) 4 h and (d) 24 h incubation.

***Relaxometry and ICP-AES***

The homogenized tissue samples were aliquoted into nuclear magnetic resonance (NMR) tubes and incubated at 37.45°C in a circulating water bath. Relaxometry data from the homogenates were collected using a benchtop time-domain NMR Minispec mq60 (Bruker) operating at 1.41 T. A Carl-Purcell-Meiboom-Gill (CPMG) spin echo sequence was used to measure T2. The echo spacing was 0.5 ms for the short TE measurements and 2 ms and for the long TE measurements. An inversion recovery (IR) sequence was used to measure T1 using 10 inversion times (TI) logarithmically spaced between 50 and 10,000 ms.

Following relaxometry measurements, all samples were acid digested and the total iron content was assessed by ICP-AES (Marine and Freshwater Research Laboratory Environmental Science, Murdoch University, Australia). Briefly, samples were transferred to pre-weighed acid digest tubes and sample weights were recorded. Concentrated nitric acid (10 ml) was added and samples were heated at 95 °C for 4-6 h (final volume ~ 1 ml). The digests were then cooled to room temperature and diluted with the addition of Milli-Q water (4 ml). Samples were weighed, then transferred to 5 ml tubes for ICP-AES analysis. The quantitation limit for Fe using ICP-AES was 0.002 mg/L.

**
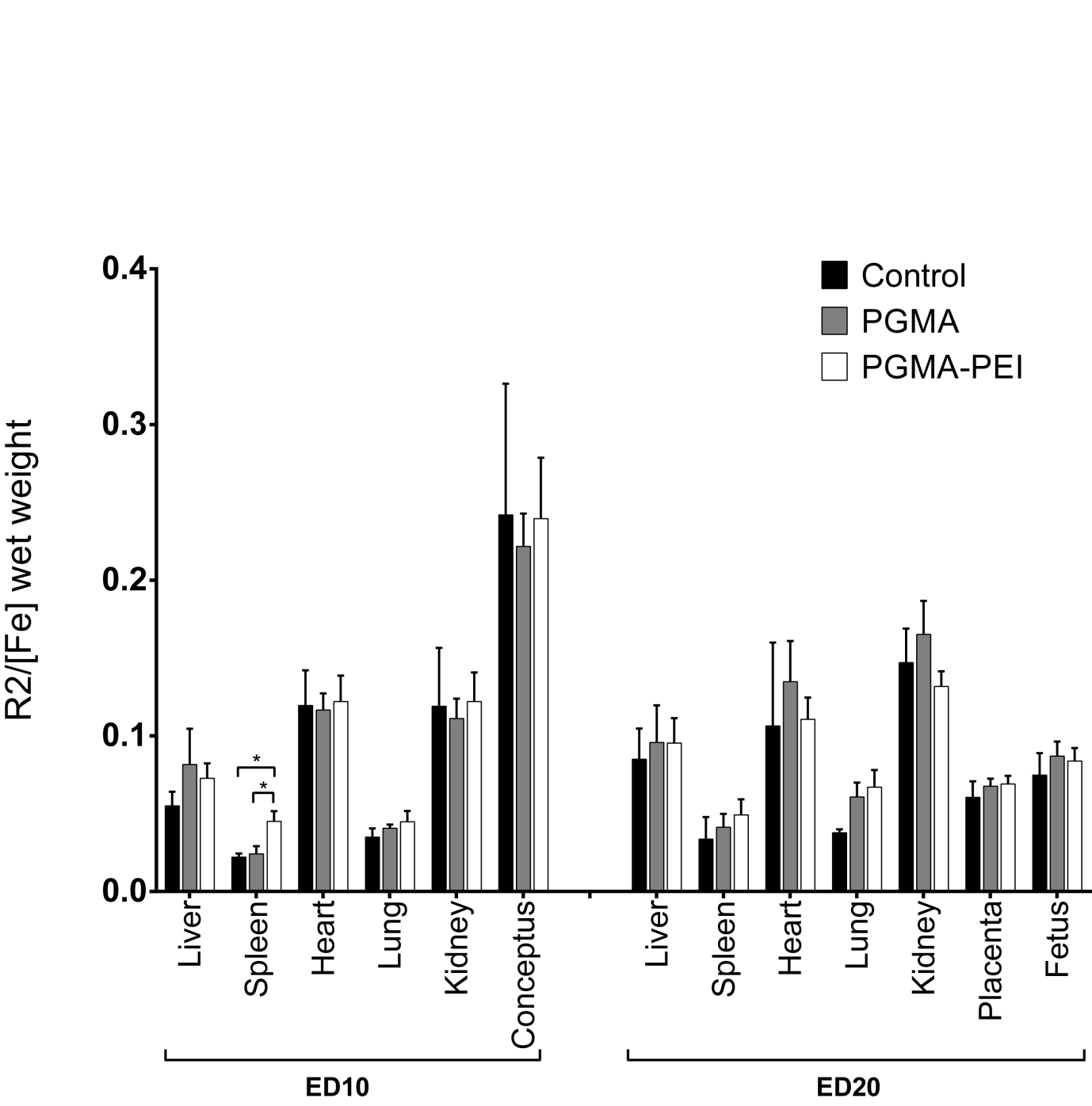
**

**Figure S2.** R2/[Fe] measurements for ED10 and ED20 maternal and fetal tissues. Significant differences in R2/[Fe] was found within the spleens of ED10 PGMA-PEI treated dams versus PGMA treated and control dams. Data reported as mean ± SD (n = 4 per group). One-way ANOVA was used for statistical analysis; *p ≤ 0.05.


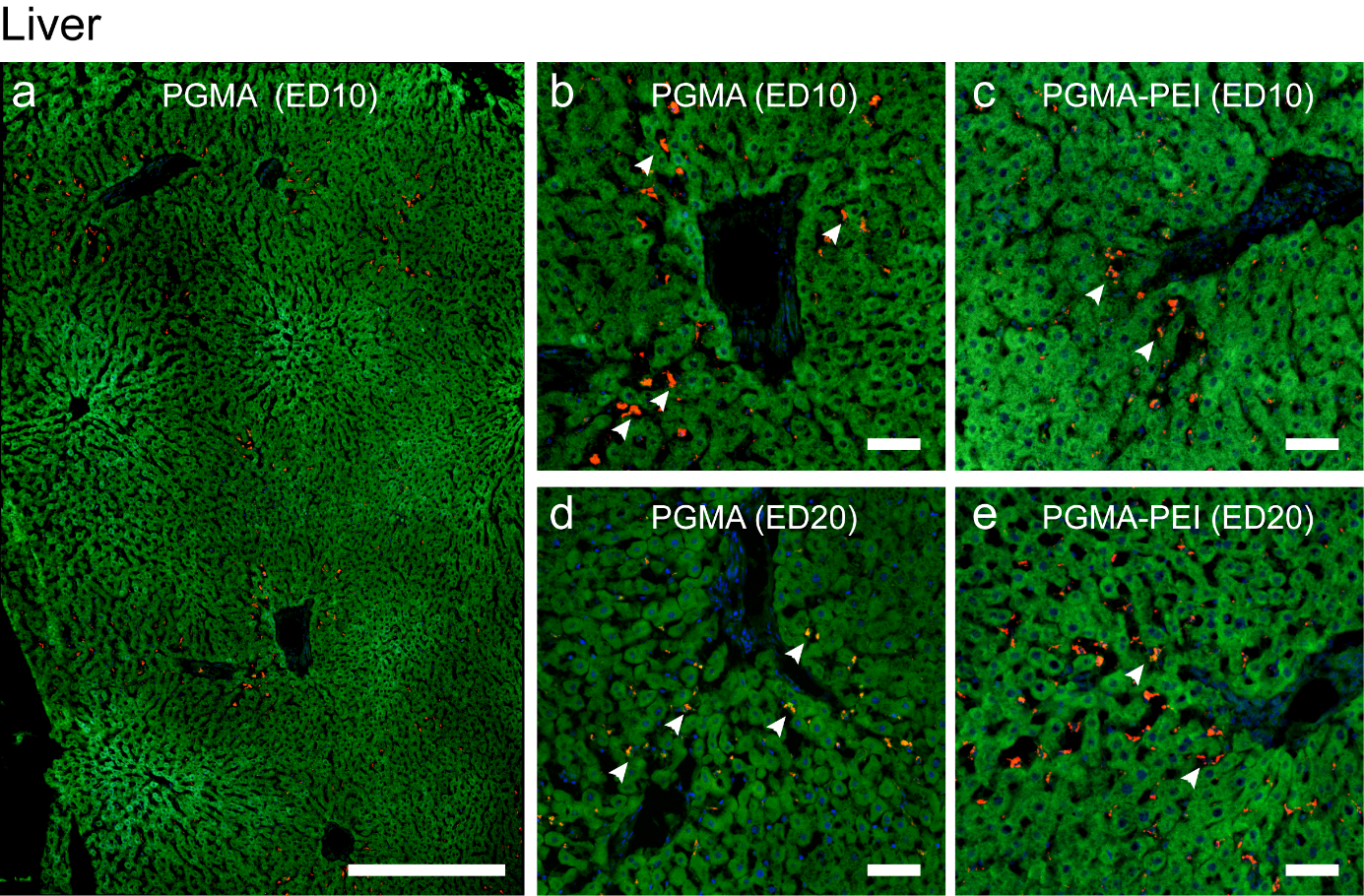


**Figure S3.** (a) Representative tissue section of a maternal liver. Higher magnification images show the accumulation of PGMA and PGMA-PEI nanoparticles in the maternal liver at ED10 (b & c) and ED20 (d & e). PGMA and PGMA-PEI nanoparticles were found to be accumulating mainly within Kupffer cells (white arrowheads) in the hepatic sinusoids of the liver, close to the central veins of the lobules. Scale bars: (a) 500 μm; (b – e) 50 μm. Images are representative of n = 4 per group.


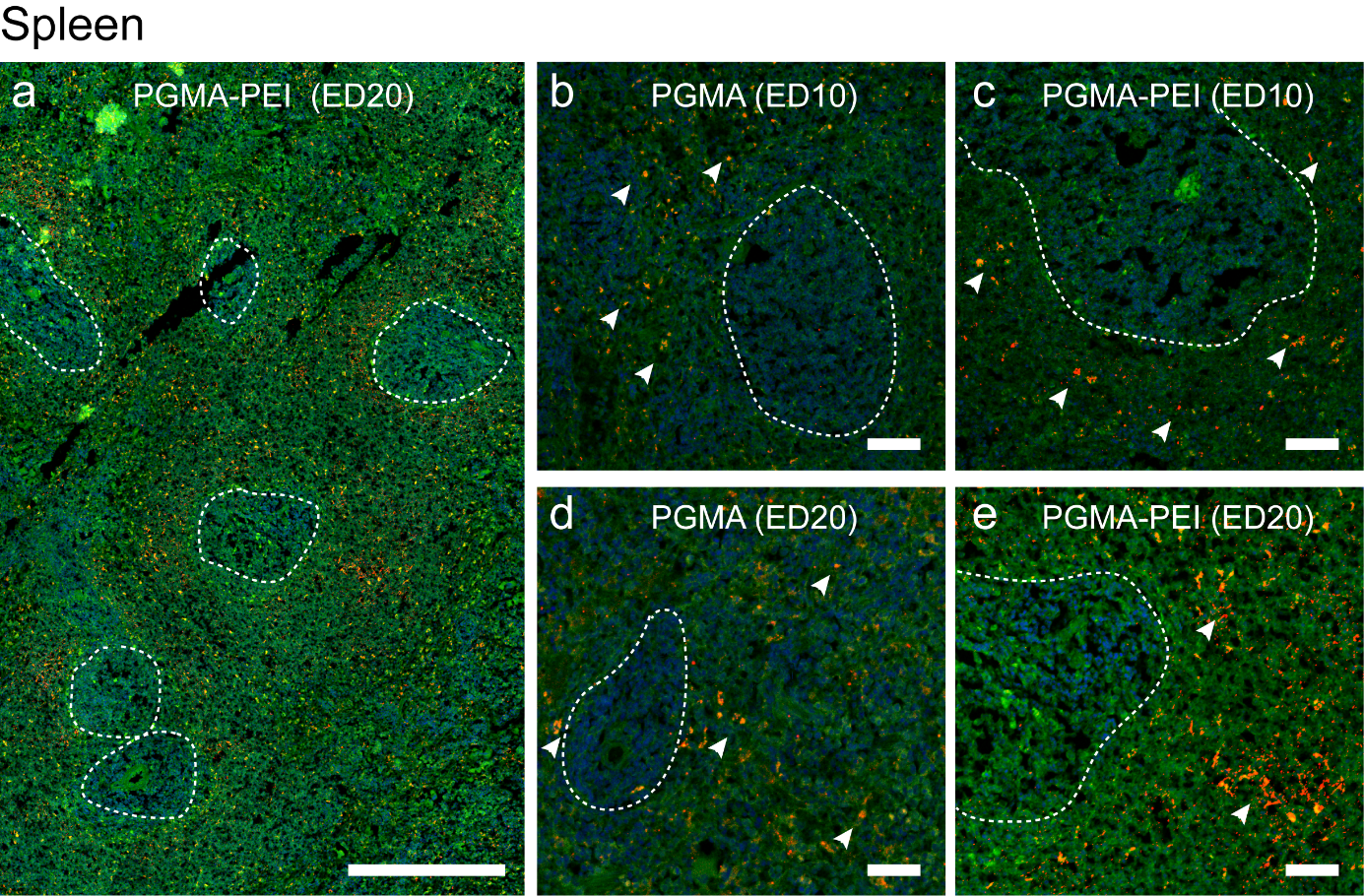


**Figure S4.** (a) Representative tissue section of a maternal spleen. Higher magnification images show the accumulation of PGMA and PGMA-PEI nanoparticles in the maternal spleen at ED10 (b & c) and ED20 (d & e). Nanoparticles (white arrowheads) were observed throughout the red pulp with higher quantities amassing around the peripheries of white pulp (denoted by dashed lines). Scale bars: (a) 500 μm; (b – e) 50 μm. Images are representative of n = 4 per group.


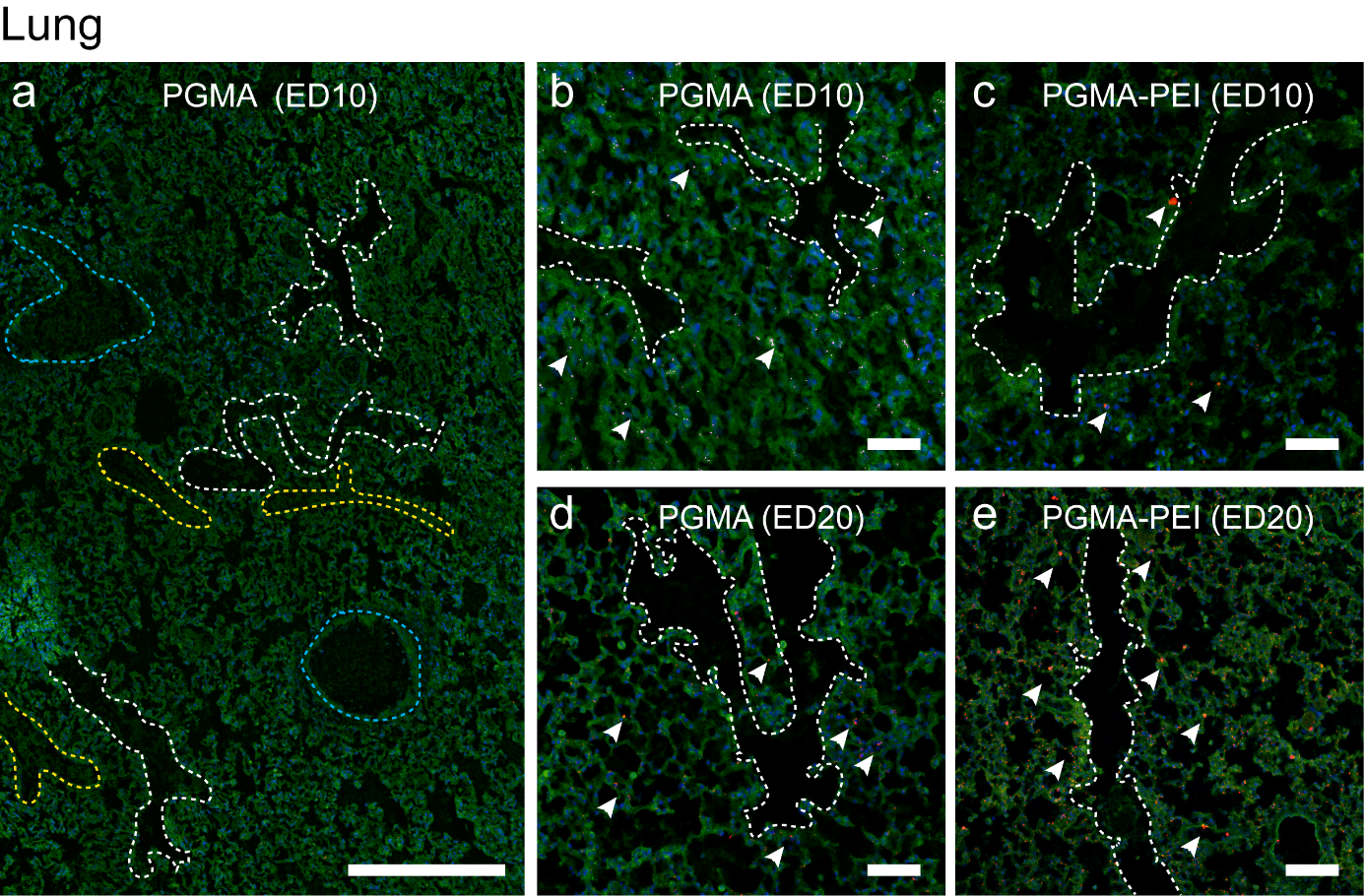


**Figure S5.** (a) Representative tissue section of a maternal lung. Higher magnification images show the accumulation of PGMA and PGMA-PEI nanoparticles in the maternal lung at ED10 (b & c) and ED20 (d & e). Widespread and even distribution of nanoparticles (white arrowheads) observed close the alveolar lining (white dashed lines) with little to none observed within the bronchioles (yellow dashed lines) and blood vessel endothelium (blue dashed lines). Scale bars: (a) 500 μm; (b – e) 50 μm. Images are representative of n = 4 per group.


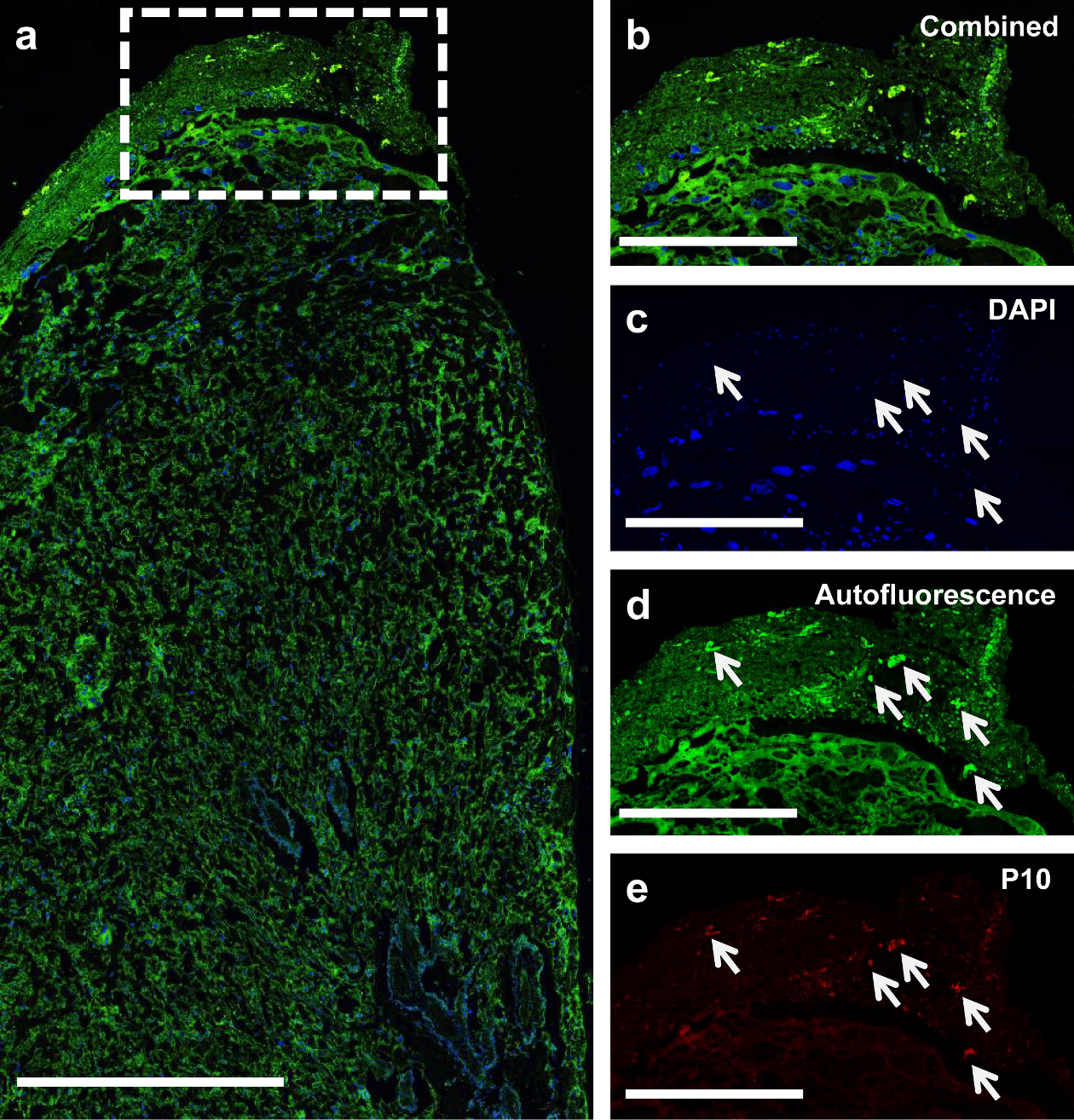


**Figure S6.** (a) Lipofuscin-like fluorescent pigments and refractile bodies at the decidual cap of a representative placenta imaged with confocal microscopy. (b-e) Magnified images consisting of the various collection channels of the highlighted area shown in (a). Fluorescent pigments were not visible in DAPI channel (λ_ex/em_ 405/425-475 nm) but visible in both autofluorescence (λ_ex/em_ 488/500-525 nm) and P10 (λ_ex/em_ 488/663-738 nm) channels. Arrows indicate the locations of prominent fluorescent pigments. Scale bar: (a) 1000 μm; (b-e) 500 μm.


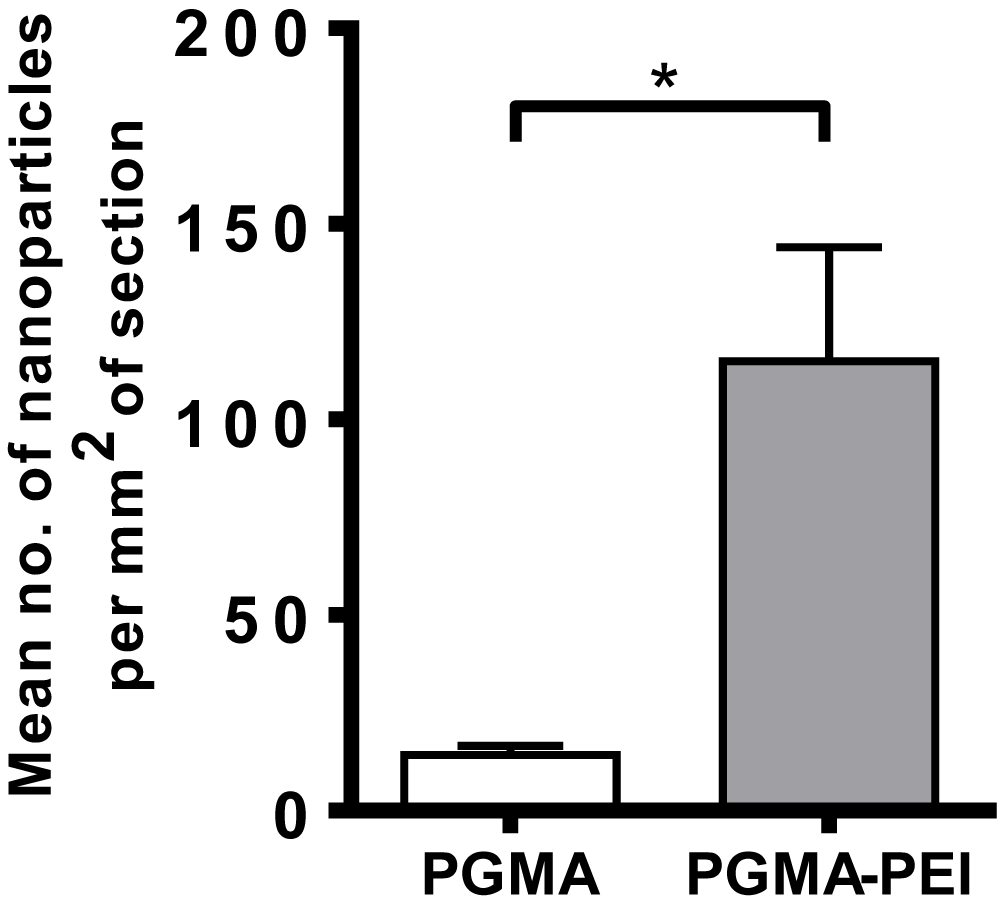


**Figure S7.** Mean number of nanoparticles counted per mm^2^ of placenta for both PGMA and PGMA-PEI treated dams. Data reported as mean ± SD(n = 4 per group). Two-tailed Student’s t-test was used for analysis; * *p* <0.05.


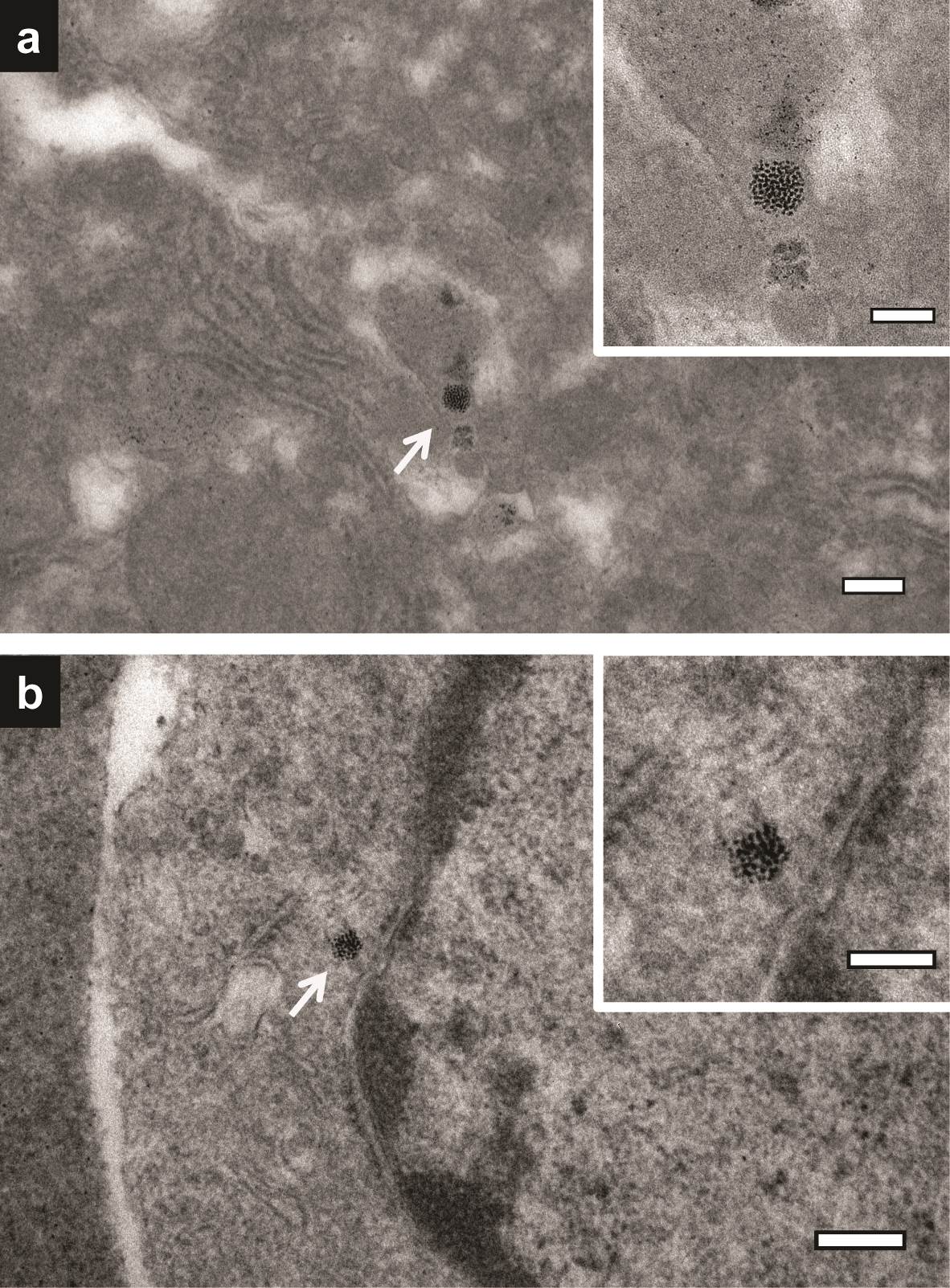


**Figure S8.** TEM images of PGMA-PEI nanoparticles in ED20 (a) maternal liver and (b) the labyrinth zone of the placenta. Arrowheads indicate location of nanoparticles within cells. Insets show the nanoparticles at high magnification. Scale bars: (a & b) 200 nm; (insets) 100 nm.
